# Disruption of Physiological Rhythms Persist Following Cessation of Cigarette Smoke Exposure in Mice

**DOI:** 10.3389/fphys.2020.501383

**Published:** 2020-10-21

**Authors:** Gilles Vanderstocken, Jade P. Marrow, Melissa A. Allwood, Martin R. Stampfli, Jeremy A. Simpson

**Affiliations:** ^1^Department of Pathology and Molecular Medicine, McMaster Immunology Research Centre, McMaster University, Hamilton, ON, Canada; ^2^Department of Medicine, Firestone Institute for Respiratory Health at St. Joseph’s Healthcare, McMaster University, Hamilton, ON, Canada; ^3^Department of Human Health and Nutritional Sciences, University of Guelph, Guelph, ON, Canada; ^4^IMPART Team Canada Investigator Network, Guelph, ON, Canada; ^5^State Key Laboratory of Respiratory Disease, Guangzhou Medical University, Guangzhou, China

**Keywords:** cigarette smoke exposure, physiological rhythms, cardiovascular disease, chronic obstructive pulmonary disease, chronotherapy

## Abstract

**Background:**

Physiological rhythms in mammals are essential for maintaining health, whereas disruptions may cause or exacerbate disease pathogenesis. As such, our objective was to characterize how cigarette smoke exposure affects physiological rhythms of otherwise healthy mice using telemetry and cosinor analysis.

**Methods:**

Female BALB/c mice were implanted with telemetry devices to measure body temperature, heart rate, systolic blood pressure (SBP), and activity. Following baseline measurements, mice were exposed to cigarette smoke for approximately 50 min twice daily during weekdays over 24 weeks. Physiological parameters were recorded after 1, 4, 8, and 24 weeks of exposure or after 4 weeks cessation following 4 weeks of cigarette smoke exposure.

**Results:**

Acute cigarette smoke exposure resulted in anapyrexia, and bradycardia, with divergent effects on SBP. Long term, cigarette smoke exposure disrupted physiological rhythms after just 1 week, which persisted across 24 weeks of exposure (as shown by mixed effects on mesor, amplitude, acrophase, and goodness-of-fit using cosinor analysis). Four weeks of cessation was insufficient to allow full recovery of rhythms.

**Conclusion:**

Our characterization of the pathophysiology of cigarette smoke exposure on physiological rhythms of mice suggests that rhythm disruption may precede and contribute to disease pathogenesis. These findings provide a clear rationale and guide for the future use of chronotherapeutics.

## Introduction

Cigarette smoking is a preventable global health epidemic, with over one billion smokers world-wide (World Health Organization, 2017). While smoking prevalence has declined since 1980, population growth has resulted in a significant increase in the number of current smokers globally (Ng et al., 2014). Approximately half of all smokers will develop serious smoking-related illness (U.S. Department of Health and Human Services et al., 2014), including cancer, cardiovascular disease (CVD), and chronic obstructive pulmonary disease (COPD) (Chaouat et al., 2008; Peinado et al., 2008; Lugade et al., 2014). While continued emphasis needs to be placed on efforts to reduce smoking prevalence, these strategies are unlikely to improve the burden of disease over the next two decades and need to be paired with a better understanding of the pathobiology of smoking-related diseases.

Exposure of animals to cigarette smoke is perceived as one of the most relevant models to investigate the pathobiology related to smoking-related diseases (Wright et al., 2008; Botelho et al., 2010; Vlahos and Bozinovski, 2014; Shu et al., 2017; Bonniaud et al., 2018). Physiological rhythms are fundamental to maintaining a healthy state, which, when lost or disrupted, cause and/or exacerbate disease (Pelissier et al., 1997, 1998; Martino et al., 2007; Voigt et al., 2016). Circadian misalignment in humans may exacerbate inflammatory responses in the lung (Yao et al., 2015), and is associated with increases in cancer (Hansen and Lassen, 2012; Wang et al., 2013), cardiometabolic disease (Guo et al., 2013) and notably, CVD (Martino et al., 2007; for review, see Truong et al., 2016). Despite this, little is known how physiological rhythms are influenced in models of cigarette smoke exposure and the role of circadian rhythm disruption as an exacerbating influence (Gebel et al., 2006; Vasu et al., 2009; Hwang et al., 2014; for review, see Sundar et al., 2015).

The aim of this study was to characterize the effects of acute and chronic cigarette smoke exposure on physiological rhythms, specifically, body temperature, heart rate, systolic blood pressure (SBP), and activity using telemetry. We found the effects of cigarette smoke exposure were immediate, showing dramatic disruption to physiological rhythms after 1 week, which persisted non-ubiquitously across 24 weeks of exposure. Four weeks of smoke exposure cessation was insufficient to allow full recovery of rhythms. Cigarette smoke exposure resulted in mixed effects on mesor, amplitude, and *R*^2^ for all physiological parameters. These observations highlight the complexity of the smoke exposure model in that cigarette smoke exposure rapidly disrupts physiological rhythms. These changes precede the development of overt pathology and may thereby promote disease pathogenesis.

## Materials and Methods

### Ethical Approval

Female BALB/c mice (Charles River Laboratories, Saint-Constant, QC, Canada) were aged 9–11 weeks, with body weights ∼19 g, prior to surgery. Mice were housed at 23°C on a 12:12 h light-dark cycle (lights on 07:00 h) under specific pathogen-free conditions and provided food and water *ad libitum*. We opted to use female mice as we have previously validated the cigarette smoke exposure model in females (Jobse et al., 2013, 2014). Following telemetry implantation, animals were housed individually in Faraday cages connected to telemetry receivers (RPC-1; Data Science International). Housing and experimental procedures were approved by the Animal Research Ethics Board at McMaster University (Hamilton, Canada) and in conformity with the guidelines of the Canadian Council on Animal Care.

### Telemetry

Telemetry surgeries were completed as previously described (Allwood et al., 2018). Briefly, mice were implanted with transmitters (HD-X11, Data Science International, St. Paul, MN, United States) equipped to measure body temperature, heart rate, SBP, and activity. Animals were anesthetized using isoflurane (2:98% isoflurane:oxygen) and body temperature was maintained at 37°C using a warmed heating pad. The telemetry unit was inserted into the abdomen and secured intraperitoneally. The pressure catheter was tunneled subcutaneously from the abdomen and secured into the right carotid artery using a 7-0 suture and vet bond (3M, London, ON, Canada). Two electrocardiography leads, one above the rib cage and the second above the abdominal wall, were secured subcutaneously to the muscle layer. Mice were administered immediate post-surgical buprenorphine and additional analgesics were provided as required. A recovery period of 2 weeks was provided to allow for restoration of normal circadian rhythms. Thereafter, baseline recordings for temperature, heart rate, SBP, and activity were acquired over 5 days in room air. Telemetry data were acquired through computer acquisition software (Dataquest ART V.3.3; Data Science International). All data were exported to Excel 2016 (Microsoft Corp., Redmond, WA, United States) for analysis and processing.

### Cigarette Smoke Exposure

Mice were exposed to cigarette smoke using a whole-body cigarette smoke exposure system (SIU-48; Promech Lab AB, Vintrie, Sweden), as described in detail previously (Botelho et al., 2010). Mice were exposed to cigarette smoke from twelve 3R4F reference cigarettes with filters removed (Tobacco and Health Research Institute, University of Kentucky, Lexington, KY, United States). Indeed, cigarettes (with filters removed) increase total particulate matter exposure (Hodge-Bell et al., 2007), which is an important driver of lung inflammation. Each cigarette was consumed in a total of 16 puffs of 10 s with a 5 s air intake time. Exposures occurred between 10:00 and 10:50 and 15:00 and 15:50 h. This exposure protocol results in carboxyhemoglobin and cotinine levels that are comparable to a human smoker (Botelho et al., 2010) and the pathological development of lung inflammation and airspace enlargement that is typical for smokers. Pre-exposure recordings took place inside the chamber for 1 h prior to exposure (i.e., 9:00–10:00 h and 14:00–15:00 h inside the chamber breathing air). Animals were returned to their housing chambers for the 3 h between exposures. This procedure was conducted only on weekdays. Mice were not exposed to cigarette smoke on weekends. Physiological responses were recorded by telemetry at baseline and on the weekend after 1, 4, 8, and 24 weeks of cigarette smoke exposure and 4 weeks of cessation.

### Sampling and Analysis

Data for body temperature, heart rate, SBP, and activity were sampled every 5 min for 30 s and averaged for each hour of the day for weekdays and the weekend. Hourly means were obtained by averaging raw data between all animals and organizing into 24 h periods (Excel 2016, Microsoft Corp., Redmond, WA, United States). Cosine wave characteristics, including mesor (i.e., an estimation of the mean statistic of circadian rhythms), acrophase (i.e., time at the cycle’s peak), and amplitude (i.e., the difference between the peak/trough and mean statistic) were evaluated using cosinor analysis as previously described (Allwood et al., 2018). *R*^2^, an index of goodness-of-fit, was also calculated using GraphPad Prism Software, version 6 (GraphPad Inc., La Jolla, CA, United States). Cosinor analysis was used to determine the best-fit sine wave using non-linear regression analysis. Differences in the best-fit values for mesor, amplitude, and acrophase were compared between experimental conditions to baseline using the extra sum-of-squares *F* test. For each parameter, twelve 1 h averages were analyzed from both light (sleep-phase) and dark (wake-phase) cycles using one-way repeated measures ANOVA. When significant differences were detected on data sets that were normally distributed, Dunnett’s *post hoc* test was performed. Further, Kruskal–Wallis analysis was used with Dunn’s *post hoc* test when data sets were non-normally distributed. Statistical analyses were performed using GraphPad Prism Software, version 6 (GraphPad Inc., La Jolla, CA, United States). A *P-*value of <0.05 was considered statistically significant.

## Results

### Cigarette Smoke Exposure Induced Hypothermia and Bradycardia

**FIGURE 1:**
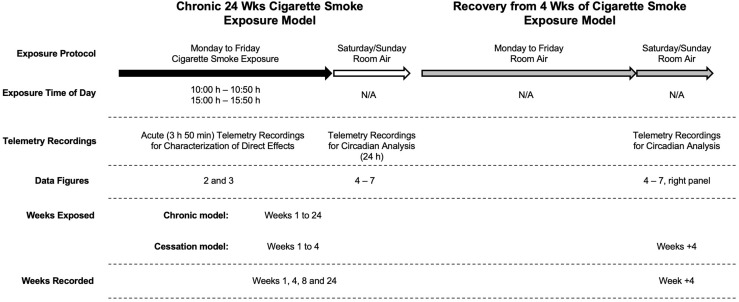
Experimental design. Cigarette smoke exposure occurred twice daily (from 10:00 h to 10:50 h and 15:00 h to 15:50 h) on weekdays (black arrow) with room air over the weekend (white arrow) for up to 24 weeks (left). In a subsequent group of mice, they followed the same cigarette smoke exposure for up to 4 weeks followed by an additional 4 weeks of room air exposure (gray arrows; right). Baseline recordings were made 1 week before exposure; sham animals were exposed to room air in the smoking chamber and used only for comparison to acute telemetry recordings.

**FIGURE 2:**
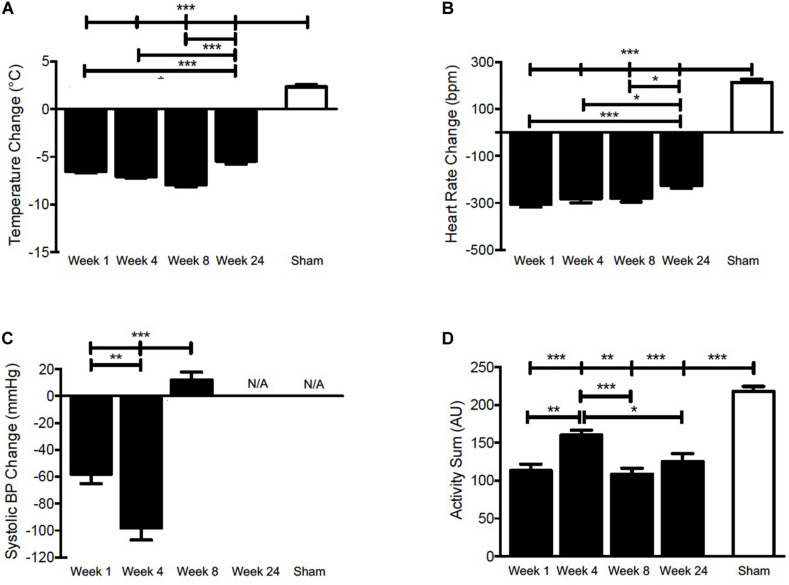
Pooled and quantified changes in acute physiological responses during 50 min CS exposure. **(A)** Body temperature, **(B)** Heart rate, **(C)** SBP, and **(D)** Activity during 50 min of cigarette smoke exposure at week 1, 4, 8, 24, or sham. The raw data can be found in Supplementary Figure 1 (doi: 10.6084/m9.figshare.9917138). Values are the mean ± SEM (*n* = 4 for each week and parameter). *indicates significance (*P* < 0.05), **indicates significance (*P* < 0.01), ***indicates significance (*P* < 0.001).

To investigate the effect of cigarette smoke exposure on physiological rhythms, female BALB/c mice were exposed to cigarette smoke for 24 weeks or 4 weeks, followed by a 4-week cessation period. Physiological responses were recorded by telemetry at baseline and on the weekend after 1, 4, 8, and 24 weeks of cigarette smoke exposure and 4 weeks of cessation. Details of the study protocol are provided in Figure 1. Figure 2 depicts the pooled and quantified changes in physiological responses during the acute exposure (i.e., 50 min). The raw data representing the complete physiological changes can be found in Supplementary Figure 1 (doi: 10.6084/m9.figshare.9917138). To compare the effects of the smoking chamber alone on acute physiological responses, sham animals were placed in the cigarette smoking chamber but were not exposed to cigarette smoke (Figure 2 and Supplementary Figure 2, doi: 10.6084/m9.figshare.12048642). During exposure, cigarette smoke caused a marked reduction in body temperature (Figure 2A). Body temperature progressively decreased during smoke exposure and returned to pre-exposure temperatures over a 3-h period following removal from the exposure box (Supplementary Figure 1, doi: 10.6084/m9.figshare.9917138).

A significant drop in body temperature was observed at weeks 1, 4, 8, and 24. Since thermogenesis is, at least in part, regulated by heart rate in murines (Swoap et al., 2004; Sack et al., 2005), we analyzed next whether the decrease in body temperature was associated with bradycardia. Figure 2B shows that heart rate decreased acutely and returned to near pre-exposure levels within 3 h following removal from the exposure box (Supplementary Figure 1, doi: 10.6084/m9.figshare.9917138), mirroring the decrease in body temperature.

### Cigarette Smoke Exposure Acutely Uncoupled the Heart Rate-Temperature Correlation

**FIGURE 3:**
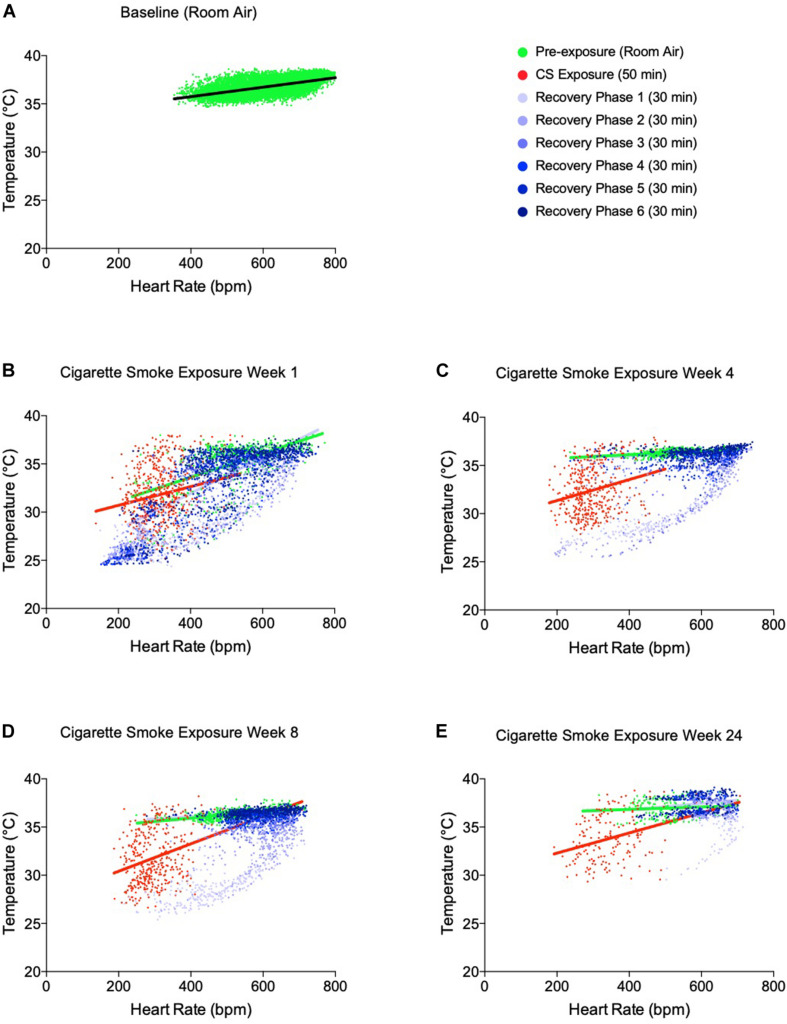
Temperature-heart rate correlation at pre-cigarette smoke exposure, during 50 min of cigarette smoke exposure and immediately post-cigarette smoke exposure (3 h of recovery at room air). **(A)** Temperature-heart rate correlation at baseline, **(B)** week 1 of cigarette smoke exposure, **(C)** week 4 of cigarette smoke exposure, **(D)** week 8 of cigarette smoke exposure, and **(E)** week 24 of cigarette smoke exposure. All colored dots represent the physiological responses of mice every 5 min with linear regression. 3 h recovery phases are separated into 30-min intervals to represent the progression of disrupted physiological responses back toward baseline.

Nicotine disrupts the linear relationship between heart rate and body temperature (Sack et al., 2005). Therefore, we next examined whether this relationship was uncoupled by exposure to cigarette smoke (Figure 3), which combined data from both daily exposure periods. Indeed, uncoupling of the heart rate-temperature mechanism occurred during the 50 min of cigarette smoke exposure for each week recorded, as compared to the hour of pre-exposure. The linear relationship between heart rate and body temperature was only partially restored within 3 h following cigarette smoke exposure. Thus, thermogenesis was compromised by bradycardia as a result of cigarette smoke exposure.

### Cigarette Smoke Exposure Had Divergent Effects on Systolic Blood Pressure

As a sympathomimetic, nicotine stimulates the sympathetic nervous system and elevates plasma norepinephrine and epinephrine (Cryer et al., 1976), thereby increasing heart rate and blood pressure in healthy individuals (U.S. Department of Health and Human Services et al., 2014; for review, see Benowitz, 2003). To confirm that the physiological response in female BALB/c mice was comparable to that typically elicited in humans, we measured SBP during cigarette smoke exposure. At the onset of exposure, there was an initial increase in SBP at weeks 1, 4, and 8 (Supplementary Figure 1, doi: 10.6084/m9.figshare.9917138).

Peak pressures were reached within 10 min of exposure and decreased thereafter at weeks 1 and 4, indicative of hypotension. A more robust hypotensive response was observed at week 4, evidenced by a greater decrease during cigarette smoke exposure (Figure 2C and Supplementary Figure 1C, doi: 10.6084/m9.figshare.9917138). Mice experienced an initial increase in SBP during the exposure period, which was maintained throughout CS exposure at week 8 (Figure 2C). Of note, SBP levels had surpassed pre-exposure levels 3 h following cigarette smoke exposure at weeks 1, 4, and 8, indicative of hypertension (Supplementary Figure 1, doi: 10.6084/m9.figshare.9917138). No SBP data are shown for week 24, as pressure recordings became inconsistent, likely as a consequence of decreased battery life, blood pressure probe slippage, or blood clot formation. Within the telemetry devices, the SBP sensors are the first to lose function due to their increased demand on battery.

### Cigarette Smoke Exposure Reduces Activity

Given that the stimulatory effects of nicotine are well-established, we sought to determine the impact of cigarette smoke on activity levels. We observed an initial increase in activity that peaked within 10 min during cigarette smoke exposure at weeks 1, 4, and 8, and 20 min at week 24 (Supplementary Figure 1, doi: 10.6084/m9.figshare.9917138). Subsequently, activity gradually decreased and returned toward baseline levels throughout the remaining cigarette smoke exposure time. Conversely, sham animals experienced a sustained increase in activity (Figure 2D), which returned to pre-exposure levels within 3 h following removal from the exposure chamber (Supplementary Figure 2, doi: 10.6084/m9.figshare.12048642). It is conceivable that an hour of residing in the smoking chamber induces an increase in activity that results from being placed in a novel environment.

Taken together, these findings show that cigarette smoke exposure has direct and profound effects on acute physiological parameters. Our next goal was to assess whether changes in physiological rhythms observed during smoke exposure would persist following smoking cessation (Figure 1).

### Cigarette Smoke Exposure and Four-Week Cessation Periods Had Non-uniform Effects on Physiological Rhythms

#### Body Temperature

**FIGURE 4:**
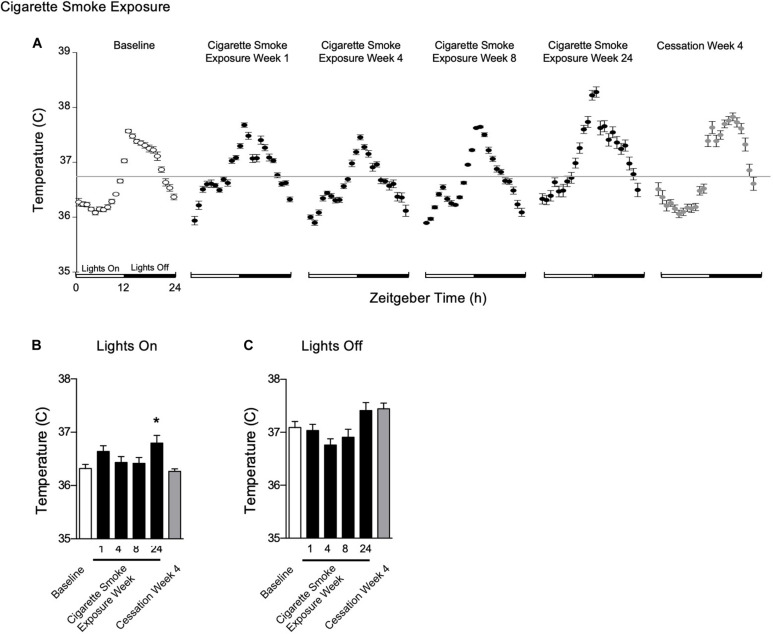
Physiological responses of body temperature at baseline, weekend 1, 4, 8, and 24 of cigarette smoke exposure, and cessation week 4. **(A)** Hourly averages for cigarette smoke exposure and cessation experiments. Horizontal gray bars represent the mesor at baseline (36.7°C). Average body temperature during **(B)** lights on and **(C)** lights off. Dunnett’s *post hoc* test was performed on normally distributed data sets. For non-normally distributed data, Kruskal–Wallis’ test was used with Dunn’s *post hoc* test. *P < 0.05 compared to baseline was considered statistically significant. All values are mean ± SEM (*n* = 9 at baseline, *n* = 4 at week 1, *n* = 4 at week 4, *n* = 5 at week 8, *n* = 5 at week 24, and *n* = 4 at cessation week 4).

To determine whether the acute fluctuations of physiological rhythms observed during cigarette smoke exposure affects mice during exposure free intervals, we analyzed body temperature, heart rate, SBP, and activity and their corresponding circadian rhythms on weekends (Supplementary Figure 3, doi: 10.6084/m9.figshare.9917168). In addition, we assessed whether physiological changes persisted following 4 weeks of cigarette smoke exposure cessation, referred henceforth as cessation (Supplementary Figure 4, doi: 10.6084/m9.figshare.12213944). After 1 week of cigarette smoke exposure, body temperature was unchanged during lights on (Figure 4B) and remained so by weeks 4 and 8. Following 24 weeks of cigarette smoke exposure, body temperature significantly increased during lights on (Figure 4A). Rhythm was mildly, yet persistently disrupted across the entire 24 weeks, as indicated by a reduction in *R*^2^ (Table 1 and Supplementary Figure 5, doi: 10.6084/m9.figshare.9917177).

**TABLE 1:** Cosinor analysis of physiological parameters following cigarette smoke exposure.

	Mesor	Amplitude	Acrophase, hours	*R*^2^
*Temperature, C*				
Baseline	36.7 ± 0.0	0.7 ± 0.0	8.6 ± 0.1	0.93
Week 1	36.8 ± 0.0*	0.5 ± 0.1*	9.0 ± 0.1*	0.81
Week 4	36.6 ± 0.0	0.5 ± 0.0*	9.1 ± 0.1*	0.86
Week 8	36.7 ± 0.0	0.6 ± 0.1	15.3 ± 0.1*	0.82
Week 24	37.1 ± 0.0*	0.8 ± 0.1	15.2 ± 0.1*	0.89
*HR, bpm*				
Baseline	582 ± 3	59 ± 4	14.6 ± 0.1	0.89
Week 1	623 ± 4*	44 ± 6*	15.0 ± 0.1*	0.75
Week 4	555 ± 5*	79 ± 7*	9.0 ± 0.1*	0.85
Week 8	564 ± 5*	78 ± 7*	9.1 ± 0.1*	0.84
Week 24	501 ± 3*	45 ± 4*	9.0 ± 0.1*	0.88
*SBP, mmHg*				
Baseline	103.2 ± 0.8	17.0 ± 1.1	2.3 ± 0.1	0.92
Week 1	119.6 ± 1.3*	6.7 ± 1.8*	9.3 ± 0.3*	0.39
Week 4	147.1 ± 1.6*	23.1 ± 2.3*	9.3 ± 0.1*	0.83
Week 8	119.8 ± 2.1*	21.9 ± 2.9	9.4 ± 0.1*	0.73
*Activity, AU*				
Baseline	3.9 ± 0.2	3.2 ± 0.3	14.6 ± 0.1	0.86
Week 1	3.4 ± 0.2*	1.8 ± 0.3*	15.0 ± 0.2*	0.63
Week 4	5.0 ± 0.3*	4.6 ± 0.5*	15.1 ± 0.1*	0.81
Week 8	4.9 ± 0.4*	4.5 ± 0.5*	15.2 ± 0.1*	0.77
Week 24	5.6 ± 0.4*	4.6 ± 0.5*	21.3 ± 0.1*	0.78

Data are mean ± SEM. SBP, systolic blood pressure; HR, heart rate; mesor, midline estimating statistic of rhythm; amplitude, half the extent of predictable variation within a cycle; acrophase, the time of overall high values recurring in each cycle; R^2^, degree of curve fit. * indicates significance (P < 0.05).

After the first week of cigarette smoke exposure, acrophase was increased, while mesor and amplitude were decreased (Table 1). By week 4, there was a decrease in amplitude and an increase in acrophase, with the latter persisting with 8 weeks of exposure (Table 1). By the end of 24 weeks of cigarette smoke exposure, mesor and acrophase were both increased (Table 1). Thus, throughout 24 weeks of cigarette smoke exposure, mesor, and amplitude responses were biphasic, while acrophase and *R*^2^ responses were monophasic but in opposite directions (Supplementary Figure 5, doi: 10.6084/m9.figshare.9917177).

Body temperature remained unchanged following 4 weeks of cessation. After 4 weeks of cessation, there was a strong agreement in *R*^2^, and mesor, amplitude, and acrophase were increased. Compared to baseline, mesor, amplitude, acrophase, and *R*^2^ responses were monophasic (Supplementary Figure 5, doi: 10.6084/m9.figshare.9917177). Thus, cigarette smoke exposure-induced anapyrexia appeared to be overcompensated for with time and through cessation.

#### Heart Rate

**FIGURE 5:**
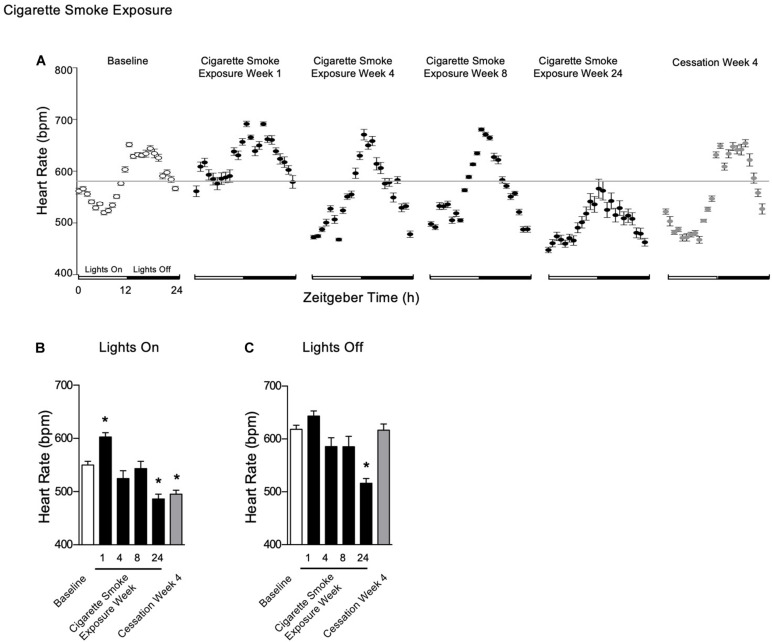
Physiological responses of heart rate at baseline, week 1, 4, 8, and 24 of cigarette smoke exposure and cessation week 4. **(A)** Hourly averages for cigarette smoke exposure and cessation experiments. Horizontal gray bars represent the mesor at baseline (584 bpm). Average heart rate during **(B)** lights on and **(C)** lights off. Dunnett’s *post hoc* test was performed on normally distributed data sets. For non-normally distributed data, Kruskal–Wallis’ test was used with Dunn’s *post hoc* test. *P < 0.05 compared to baseline was considered statistically significant. All values are mean ± SEM (*n* = 9 at baseline, *n* = 4 at week 1, *n* = 4 at week 4, *n* = 5 at week 8, *n* = 5 at week 24, and *n* = 4 at cessation week 4).

After 1 week of cigarette smoke exposure, heart rate was increased during lights on, however, by 24 weeks, it was decreased during both lights on and off (Figures 5A–C). By 1 week of exposure, amplitude was decreased, while mesor and acrophase were increased (Table 1). After 4 and 8 weeks of cigarette smoke exposure, amplitude was increased, while mesor and acrophase were decreased (Table 1). At 24 weeks, amplitude was decreased, while the decrease in mesor and acrophase persisted (Table 1). Thus, throughout 24 weeks of cigarette smoke exposure, amplitude was triphasic, *R*^2^ was monophasic, and mesor and acrophase responses were biphasic (Supplementary Figure 5, doi: 10.6084/m9.figshare.9917177). Finally, there was a mild disagreement in *R*^2^ at all time points studied (Table 1).

After 4 weeks of cessation, heart rate was decreased during lights on, whereas it was unchanged during lights off (Figures 5B,C). There was a strong agreement in *R*^2^, while mesor was decreased and amplitude was increased (Table 2). Further, there was a mild disagreement in *R*^2^ by the end of the cessation period (Table 2). Compared to baseline, mesor and amplitude were monophasic (in opposite directions), while acrophase and *R*^2^ were biphasic (Supplementary Figure 5, doi: 10.6084/m9.figshare.9917177). Thus, mice became bradycardic with chronic cigarette smoke exposure. Cigarette smoke exposure cessation appeared to ameliorate the rhythmic disruptions.

**TABLE 2:** Cosinor analysis of physiological parameters following cessation of cigarette smoke exposure.

	Mesor	Amplitude	Acrophase, hours	*R*^2^
*Temperature, C*				
Baseline	36.7 ± 0.0	0.7 ± 0.0	8.6 ± 0.1	0.93
Week 4 CSE	36.6 ± 0.0	0.5 ± 0.0*	9.1 ± 0.1*	0.86
Week 4 Cessation	36.9 ± 0.0*	0.9 ± 0.1*	20.7 ± 0.1*	0.93
*HR, bpm*				
Baseline	582 ± 3	59 ± 4	14.6 ± 0.1	0.89
Week 4 CSE	555 ± 5*	79 ± 7*	9.0 ± 0.1*	0.85
Week 4 Cessation	556 ± 4*	94 ± 5*	20.8 ± 0.1	0.94
*SBP, mmHg*				
Baseline	103.2 ± 0.8	17.0 ± 1.1	2.3 ± 0.1	0.92
Week 4 CSE	147.1 ± 1.6*	23.1 ± 2.3*	9.3 ± 0.1*	0.83
Week 4 Cessation	126.5 ± 1.4*	23.4 ± 1.9*	14.5 ± 0.1*	0.88
*Activity, AU*				
Baseline	3.9 ± 0.2	3.2 ± 0.3	14.6 ± 0.1	0.86
Week 4 CSE	5.0 ± 0.3*	4.6 ± 0.5*	15.1 ± 0.1*	0.81
Week 4 Cessation	5.3 ± 0.4*	5.2 ± 0.6*	20.5 ± 0.1	0.76

Data are mean ± SEM. SBP, systolic blood pressure; HR, heart rate; mesor, midline estimating statistic of rhythm; amplitude, half the extent of predictable variation within a cycle; acrophase, the time of overall high values recurring in each cycle; R^2^, degree of curve fit. * indicates significance (P < 0.05).

#### Systolic Blood Pressure

**FIGURE 6:**
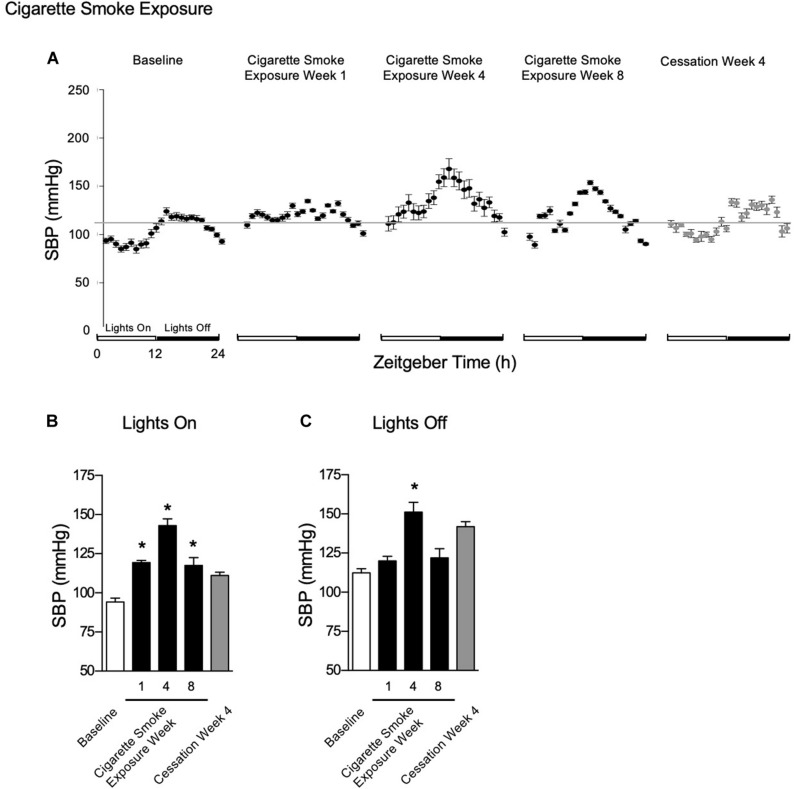
Physiological responses of SBP at baseline, week 1, 4, and 8 of cigarette smoke exposure, and cessation week 4. **(A)** Hourly averages for cigarette smoke exposure and cessation experiments. Horizontal gray bars represent the mesor at baseline (103.2 mmHg). Average SBP during **(B)** lights on and **(C)** lights off. Dunnett’s *post hoc* test was performed on normally distributed data sets. For non-normally distributed data, Kruskal–Wallis’ test was used with Dunn’s *post hoc* test. *P < 0.05 compared to baseline was considered statistically significant. All values are mean ± SEM (*n* = 7 at baseline, *n* = 4 at week 1, *n* = 3 at week 4, *n* = 3 at week 8, *n* = 2 at week 24, and *n* = 2 at cessation week 4).

One, four, and eight weeks of cigarette smoke exposure (Figure 6A) resulted in an increase in SBP during lights on (Figure 6B). Further, SBP was increased during lights off after 4 weeks of cigarette smoke exposure (Figure 6C). After 1 week of cigarette smoke exposure, there was a strong disagreement in *R*^2^, with a decrease in amplitude and increase in mesor and acrophase (Table 1). After 4 weeks of cigarette smoke exposure, there was a mild disagreement in *R*^2^, and mesor, amplitude, and acrophase were all increased (Table 1). A mild disagreement in *R*^2^ and increases in mesor and acrophase persisted after 8 weeks of cigarette smoke exposure (Table 1). Thus, throughout 8 weeks of cigarette smoke exposure, amplitude was biphasic, whereas mesor, acrophase, and *R*^2^ responses were monophasic (Supplementary Figure 5, doi: 10.6084/m9.figshare.9917177).

After 4 weeks of cessation, SBP was increased during lights on and off, which returned to baseline pressures by 8 weeks of cigarette smoke exposure cessation (Figures 6B,C). After 4 weeks of cessation, *R*^2^ responses trended toward a strong agreement and mesor, amplitude, and acrophase were increased (Table 2). Compared to baseline, mesor, amplitude, acrophase, and *R*^2^ responses were monophasic (Supplementary Figure 5, doi: 10.6084/m9.figshare.9917177). Thus, cigarette smoke exposure-induced hypertension is, at least in part, resolved through cessation.

The percent of nocturnal blood pressure dip is an important clinical consideration. Indeed, loss of circadian SPB rhythm in patients is defined as the difference between active-phase mean SBP and sleep-phase mean SBP, as a percentage of active-phase SBP, where dips of less than 10% are considered predictors of future adverse cardiovascular events (Bloomfield and Park, 2015). Dips between 10 and 20% are referred to as “normal,” while above 20% is considered exaggerated. We sought to determine the time-course whereby mice exposed to chronic CS exposure experienced “non-dipping” events. At baseline, percent dip was 16%; by week 1 post-CS, the percent dip decreased to 0.6%. Thereafter, the percent dip remained below 10% at weeks 4 and 8 post-CS (5 and 4%, respectively). However, by 4 weeks of cessation, the nocturnal percent dip had increased to an acceptable level of 22%. These data would suggest that CS exposure induced the “non-dipping” phenomenon that is observed in humans who have lost SBP circadian rhythmicity, but that the rhythm was recovered with 4 weeks of cessation.

#### Activity

**FIGURE 7:**
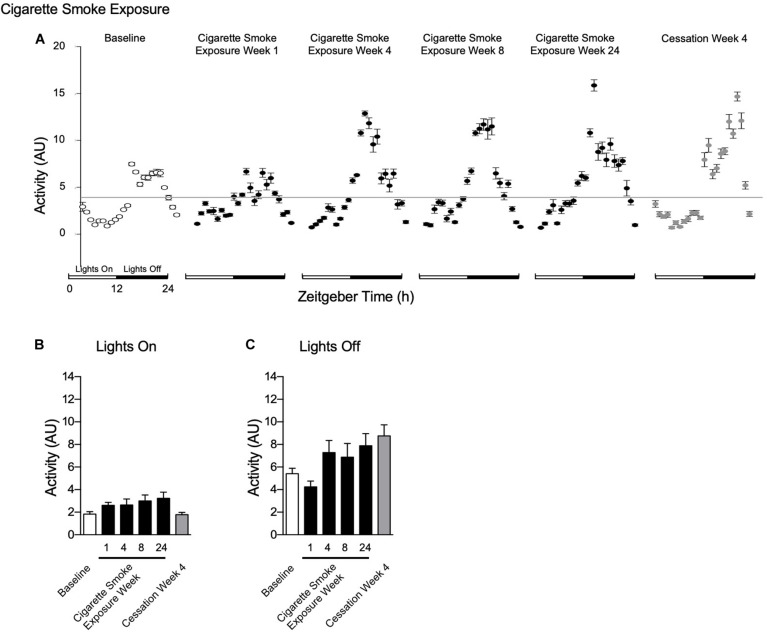
Physiological responses of activity at baseline, week 1, 4, 8, and 24 of cigarette smoke exposure, and cessation week 4. **(A)** Hourly averages for cigarette smoke exposure and cessation experiments. Horizontal gray bars represent the mesor at baseline (3.6 AU). Average activity during **(B)** lights on and **(C)** lights off. Dunnett’s *post hoc* test was performed on normally distributed data sets. For non-normally distributed data, Kruskal–Wallis’ test was used with Dunn’s *post hoc* test. *P* < 0.05 compared to baseline was considered statistically significant. All values are mean ± SEM (*n* = 9 at baseline, *n* = 4 at week 1, *n* = 4 at week 4, *n* = 5 at week 8, *n* = 5 at week 24, and *n* = 4 at cessation week 4).

Activity levels were unchanged during lights on and off throughout 24 weeks of cigarette smoke exposure (Figures 7A–C). After 1 week of cigarette smoke exposure, there was a strong disagreement in *R*^2^, while mesor, amplitude, and acrophase were decreased (Table 1). After weeks 4, 8, and 24 of cigarette smoke exposure, there was a mild disagreement in *R*^2^, whereas mesor, amplitude, and acrophase were increased (Table 1). Thus, throughout 24 weeks of cigarette smoke exposure, mesor, amplitude, and acrophase responses were biphasic, while *R*^2^ was monophasic (Supplementary Figure 5, doi: 10.6084/m9.figshare.9917177).

The mild disagreement in *R*^2^ continued following 4 weeks of cessation, and mesor and amplitude were increased (Table 2). In comparison to baseline, mesor, amplitude, and *R*^2^ responses were monophasic, whereas acrophase was biphasic (Supplementary Figure 5, doi: 10.6084/m9.figshare.9917177).

## Discussion

Here we show a temporal pathophysiological map of the physiological rhythms for body temperature, heart rate, SBP, and activity in response to acute and chronic cigarette smoke exposure and following cessation of smoke exposure in female mice. Acute cigarette smoke exposure caused bradycardia and profound hypothermia with divergent effects on blood pressure, which recovered following removal from the exposure chamber. Long term, cigarette smoke exposure caused progressive changes in heart rate, blood pressure, body temperature, and activity. While 24 h rhythmicity was largely retained, the individual wave characteristics were progressively distorted throughout the study for all measured parameters. Of note, the pathophysiological effects of cigarette smoke exposure (4 weeks) were not fully recoverable following 4 weeks of cessation. Further, we observed the effects of cigarette smoke exposure with 8 weeks cessation in two mice (data not shown). Indeed, physiological disruptions had not recovered during this prolonged cessation period. Together, these salient findings reinforce the complexity of cigarette smoke exposure pathophysiology, wherein distortion of physiological rhythms preceded the onset of overt lung disease in this model (Jobse et al., 2013, 2014). Whether perturbations in physiological rhythms are, at least in part, contributing to disease development remains to be investigated. As the cigarette smoke exposure pathophysiology causes specific and progressive perturbation in physiological rhythms, it follows that the underlying cellular and molecular mechanism(s) will also have distinct circadian patterns, which merit further investigation.

In our study, cigarette smoke exposure caused divergent blood pressure responses. The chronic effects of cigarette exposure in our mice (i.e., 8 weeks) aligns with the well-known hypertensive effects of cigarettes reported in humans (Gao et al., 2017). Surprisingly, in the previously unexposed mouse (i.e., naïve lungs to cigarette smoke exposure), significant hypotension was observed (i.e., week 1 and 4), however, whether cigarettes induce hypotension in naïve humans (i.e., non-smokers) is unknown. Cigarette smoke increases blood carboxyhemoglobin levels (CoHb, carbon monoxide and Hb) (Watson et al., 1987), resulting in hypoxia (Fricker et al., 2018). Our findings agree with previous work (Steiner et al., 2000; Allwood et al., 2018) on hypoxia-induced anapyrexia (i.e., a decrease in thermoneutral body temperature), bradycardia, and hypotension in mice. While we observed a greater decrease in SBP in response to cigarette exposure compared to traditional hypoxic hypoxia, it is likely due to the combined hypoxia and confounding effects of the thousands of chemicals in cigarette smoke. To our knowledge, we are the first to report the immediate effects of cigarette smoke exposure on activity levels in mice. As the reduction in activity levels were paralleled by decreases in body temperature, heart rate, and blood pressure, these data suggest that the physiological adaptions induced by cigarette smoke modulate the subsequent activity level (for review, see Gu and Jun, 2018).

A strong correlation between heart rate and temperature was observed prior to cigarette smoke exposure; however, this linear relationship was disrupted during cigarette smoke exposure (Figure 4). A closer look at the individual responses of heart rate and temperature revealed that cigarette smoke exposure-induced anapyrexia was only apparent during acute exposure, with temperatures returning to baseline levels within 2 h following exposure. At the same time, heart rate was also reduced, indicating it was no longer responsible for regulating temperature homeostasis (Figure 3). Nicotine-induced hypothermic responses occur in rats (Knox et al., 1973), felines (Hall, 1972), and non-human primates (Hall and Myers, 1971, 1972). Quite possibly, nicotine in cigarette smoke stimulated comparable responses in our model. Sack et al. (2005) showed that nicotine infusion-induced thermo-reductions in β4 null mice were coupled with an increase in heart rate, suggesting tachycardia was initiated as a compensatory mechanism to maintain temperature homeostasis (i.e., however, presenting as a failed compensatory mechanism of restoring body temperature). In contrast, their wildtype controls and α5- and α7-null mice experienced hypothermia accompanied by bradycardia, similar to our study. While we agree that the heart rate-mediated thermogenesis mechanism can be acutely uncoupled, Sack et al. (2005) further speculate that this response is mediated by genetic factors in mice. Accordingly, studies should elucidate the effects of cigarette smoke exposure-induced anapyrexia in different mouse strains. The long-term health effects of dismantling this mechanism remain unclear yet merits further investigation.

Whether physiological rhythm disruptions were the result of cigarette smoke exposure-induced clock gene dysfunction remains to be fully elucidated. Our observations were exclusively based upon telemetry and subsequent cosinor analysis of rhythms, thus no molecular investigations were performed. By consequence, we cannot comment on the molecular mechanisms by which cigarette smoke exposure disrupted the rhythms of body temperature, heart rate, SBP, or activity. The effects of cigarette smoke on rhythms may have been confounded by brief interruptions to sleep as smoke exposure occurred during their sleep-phase (i.e., daytime). Nevertheless, this experimental protocol is commonly used and thus, consistently reflects the majority of the investigations in animal models with cigarette exposure (Pelissier et al., 1997, 1998; Hwang et al., 2014; Bonniaud et al., 2018). We acknowledge that our findings in mice do not necessarily recapitulate COPD symptoms in humans, where interindividual, time of day, and day-to-day symptom variability are common features (Kessler et al., 2011). While murine is one of the most common research species for modeling disease, translation of findings to humans can be challenging given the large differences in metabolic rate that can affect pharmacokinetics and allometric scaling (for reviews, see Huang and Riviere, 2014; Nair and Jacob, 2016). However, our data may enhance our understanding of the pathophysiology of cigarette smoking-associated diseases and provides a time-course that may be particularly useful in future studies of chronotherapy.

Growing evidence suggests cigarette smoking severely affects circadian rhythms, which may further contribute to its role in the cause and disease progression of COPD (for review, see Truong et al., 2016; Krakowiak and Durrington, 2018). Although the evidence is not currently well-established, it appears that cigarette smoke targets a multitude of COPD-related symptoms, which normally display their own circadian oscillations and vary in severity throughout the day. For example, exacerbation of mucus secretion and excessive coughing at Zeitgeber Time 04:00 h (i.e., early morning) are observed (Barnes, 1985; for review, see Sundar et al., 2015; Krakowiak and Durrington, 2018). Maintaining the coordination between the endogenous circadian clocks and exogenous light/dark cycles is pertinent for optimizing all aspects of mammalian physiology (for review, see Bechtold et al., 2010; Truong et al., 2016). Shift work, hospital settings, and cigarette smoke exposure are environmental stimuli which initiate internal desynchrony between the central and peripheral clocks and is harmful to one’s health (for review, see Truong et al., 2016). Interestingly, shift workers are more likely to smoke compared to day workers (Ramin et al., 2015). For this reason, the interplay between both smoking and shift work may contribute to circadian rhythm disruption, however, their relative contributions remain to be determined. This misalignment is associated with hastening the development and inducing exacerbations of many pathological conditions, including inflammation, lung dysfunction, sleep and metabolic disorders, cancer, CVD and COPD (Scheer et al., 2009; for review, see Truong et al., 2016). In rodent lungs, cigarette smoke exposure alters the timing and amplitude of various clock genes, thereby affecting many downstream mechanical and physiological processes (Gebel et al., 2006; Hwang et al., 2014). Our results show alterations in timing and amplitude of physiological rhythms as early as the first week of exposure (Table 1). Accordingly, cigarette smoke exposure-induced clock dysfunction is an instigator for molecular and cellular alterations that culminate into pathophysiological lung function, which persists throughout the pathogenesis of COPD. Thus, we have shown the pathophysiology of cigarette smoke exposure on the physiological rhythms in mice, which provides proof-of-concept that circadian disturbances precede the onset and may facilitate the progression of COPD (Table 1). Upon resynchronization, the adverse effects on cardiovascular health are attenuated (i.e., the normal compensatory hypertrophic responses were reinstated in the phenotype) (Martino et al., 2007). Therefore, perhaps the reversal of abnormal pathophysiology can be achieved via restoration of circadian rhythms.

An important limitation to consider is that a circadian marker was not used in this study. Thus, any shifts to the acrophases of body temperature, heart rate, SBP, and activity could not be accounted for in the Lights On/Off mesor data presented in Figures 4, 7B,C. Second, we performed our experiments in female BALB/c mice, which are known to have different circadian pacemaker properties compared to the other commonly used inbred mouse strain, C57BL/6 (Schwartz and Zimmerman, 1990). In fact, the two strains have non-pathological disparities in their free-running period of the circadian pacemaker (when measured in constant environmental darkness), suggesting that differences in genetic background affect circadian rhythmicity (Schwartz and Zimmerman, 1990). Thus, we recommend that future studies consider these interstrain differences when designing and interpreting their results. Further, our experiments were only conducted in female mice. Comorbidities associated with smoking, including COPD, mental health, heart failure, cancer, metabolic disease, among many others, vary by sex and gender. For instance, compared to men, women who are occasional smokers, or previously were smokers, are at a higher risk for depression (Husky et al., 2008). Further, evidence suggests that women are more likely to experience worse adverse effects of tobacco from cigarette smoking compared to men, often presenting with more severe diseases (e.g., COPD) despite similar smoking history (Gan et al., 2006; Martinez et al., 2007). Therefore, while sex-specific responses to cigarette smoke exposure in animal models are limited, translating our work to humans should consider sex/gender-specific analyses, as comorbidities associated with smoking varies by gender.

While cigarette smoke is a complex mixture of particulates and many biological active chemicals, we have not separated or determined which component(s) are the cause and mechanism of physiological rhythm disruption (i.e., nicotine, carbon monoxide, total particulate matter, or processes occurring secondary to the exposure (e.g., stress, anxiety, hypoxia, hypercapnia)).

## Conclusion

Disturbances to circadian rhythms adversely affect organ function. Our research shows cigarette smoke exposure induces a variety of alterations to physiological parameters, both acutely and chronically. We demonstrate that early disruption to physiological rhythms after just 1 week precedes disease pathogenesis beyond 24 weeks of exposure. Cigarette smoke clearly has more than just a direct effect on target organs. Therefore, cigarette smoke exposure-induced circadian misalignment may be a contributing factor in the pathogenesis of a variety of diseases. Indeed, restoration of circadian rhythms may be the necessary component in arresting the development of unfavorable phenotypes and to restore proper organ function. Future research must consider the direct effects of cigarette smoke on circadian rhythms to create a new avenue of therapies where time-of-day administration of drug treatments are considered and their effectiveness at alleviating disease-related symptoms are improved.

## Data Availability Statement

All datasets generated for this study are included in the article/Supplementary Material.

## Ethics Statement

The animal study was reviewed and approved by the Animal Research Ethics Board at McMaster University (Hamilton, Canada).

## Author Contributions

GV, MS, and JS: conception and design. GV and MA: acquisition of data. JM, GV, MA, MS, and JS: analysis and interpretation, and drafting the manuscript and intellectual input. All authors approved the final version to be published.

## Conflict of Interest

The authors declare that the research was conducted in the absence of any commercial or financial relationships that could be construed as a potential conflict of interest.

## Abbreviations

COPD: chronic obstructive pulmonary disease
CVD: cardiovascular disease
SBP: systolic blood pressure.
