# Supplementary figures and images for: Disruption of Physiological Rhythms Persist Following Cessation of Cigarette Smoke Exposure in Mice
Source: Front Physiol. 2020 Oct 21;11:501383. doi: 10.3389/fphys.2020.501383 (PMC7609783; doi:10.3389/fphys.2020.501383)

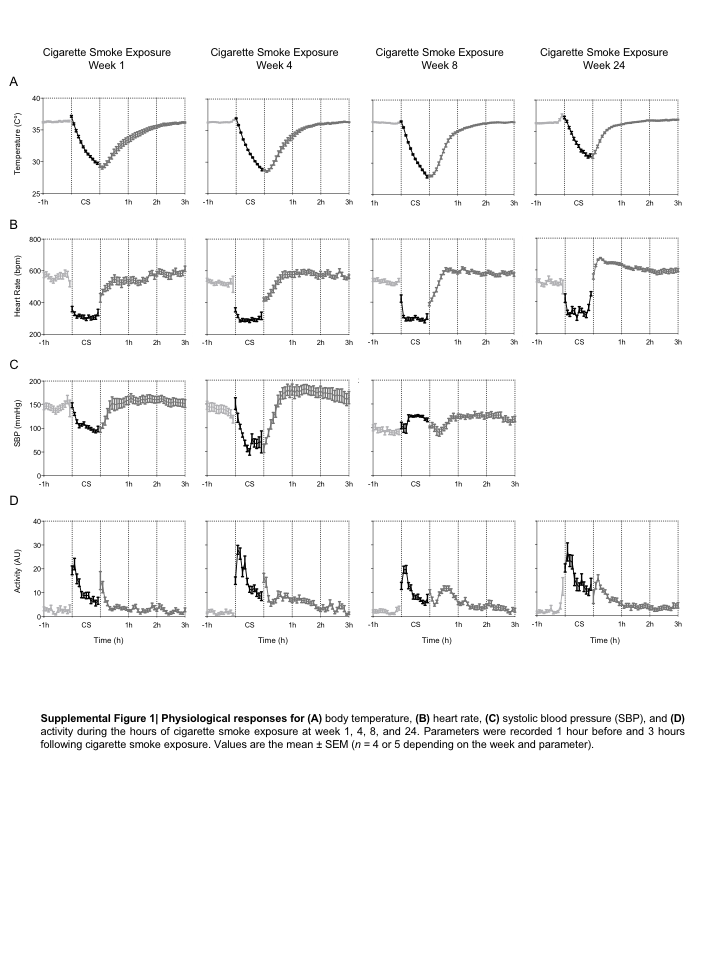

Supplement: Supplementary file 1 [file Image_1.TIFF]

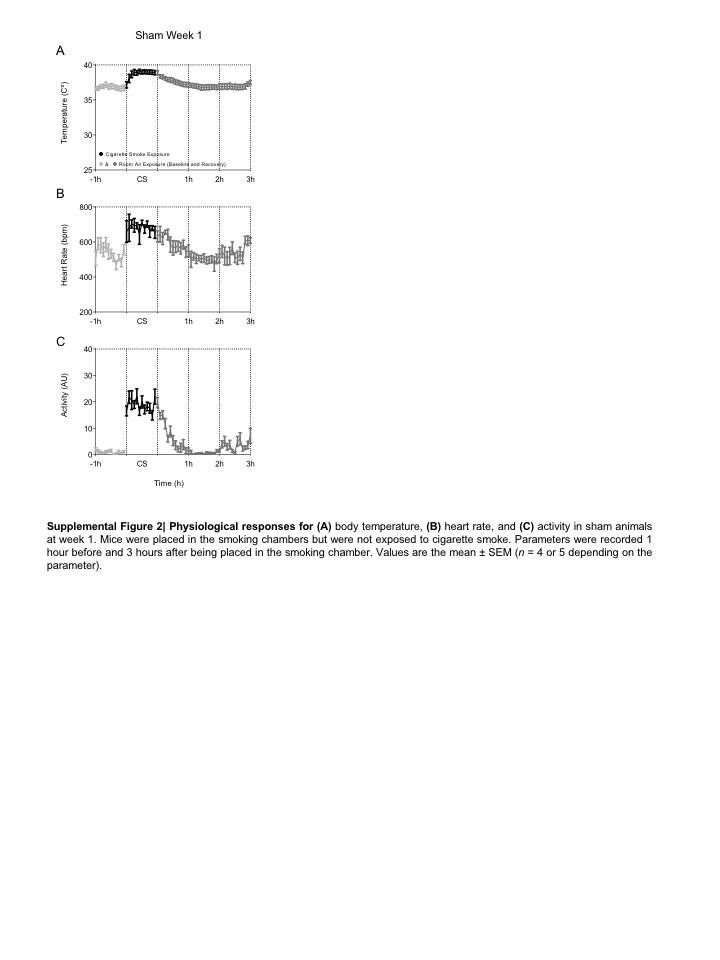

Supplement: Supplementary file 2 [file Image_2.TIFF]

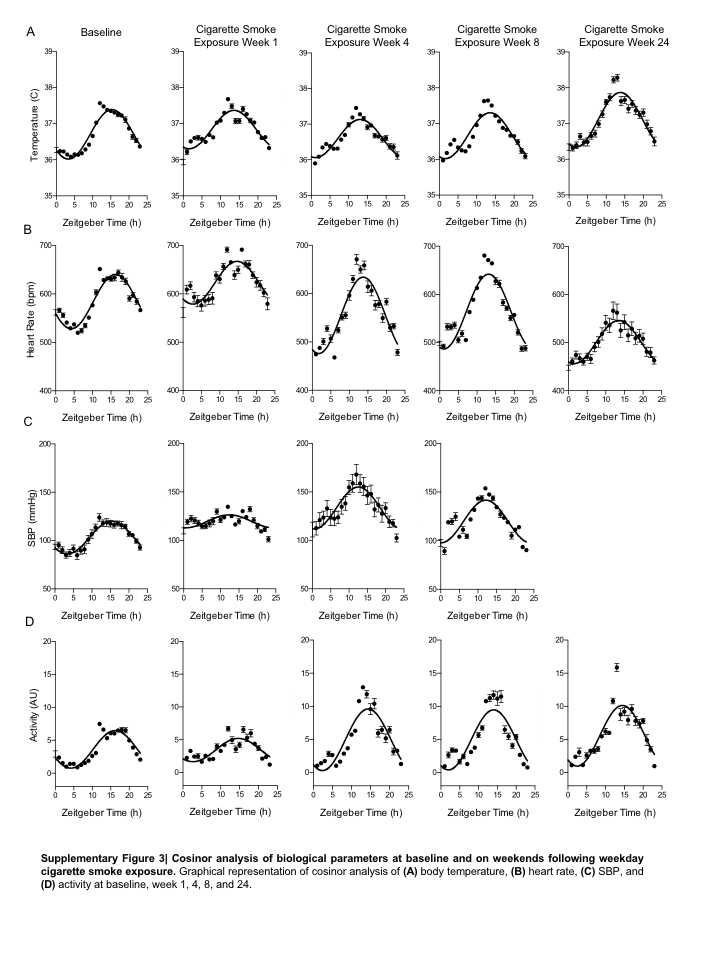

Supplement: Supplementary file 3 [file Image_3.TIFF]

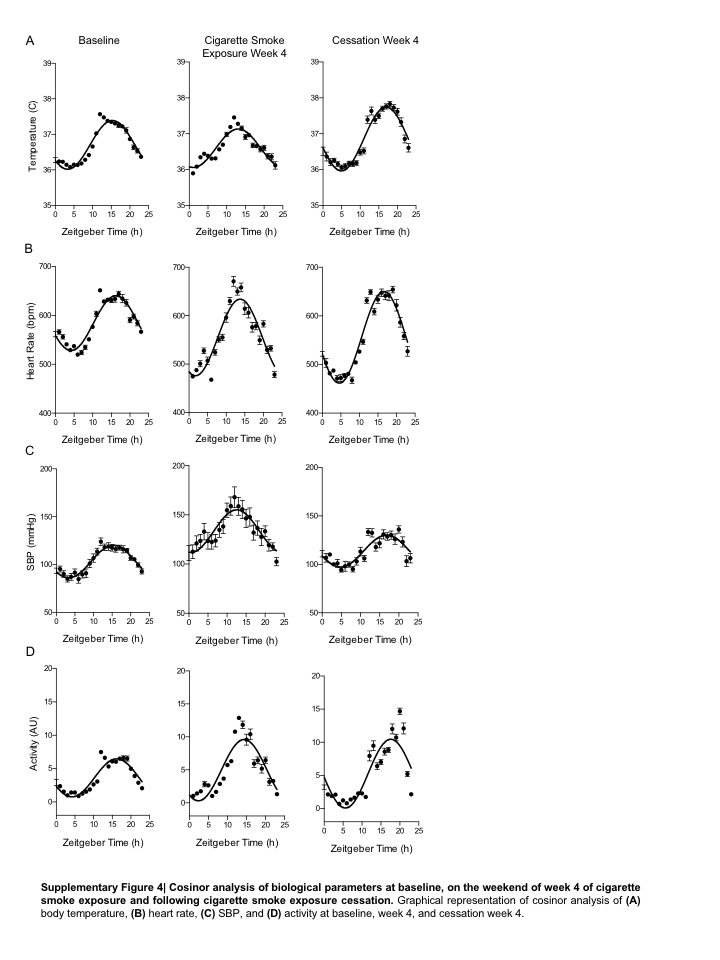

Supplement: Supplementary file 4 [file Image_4.TIFF]

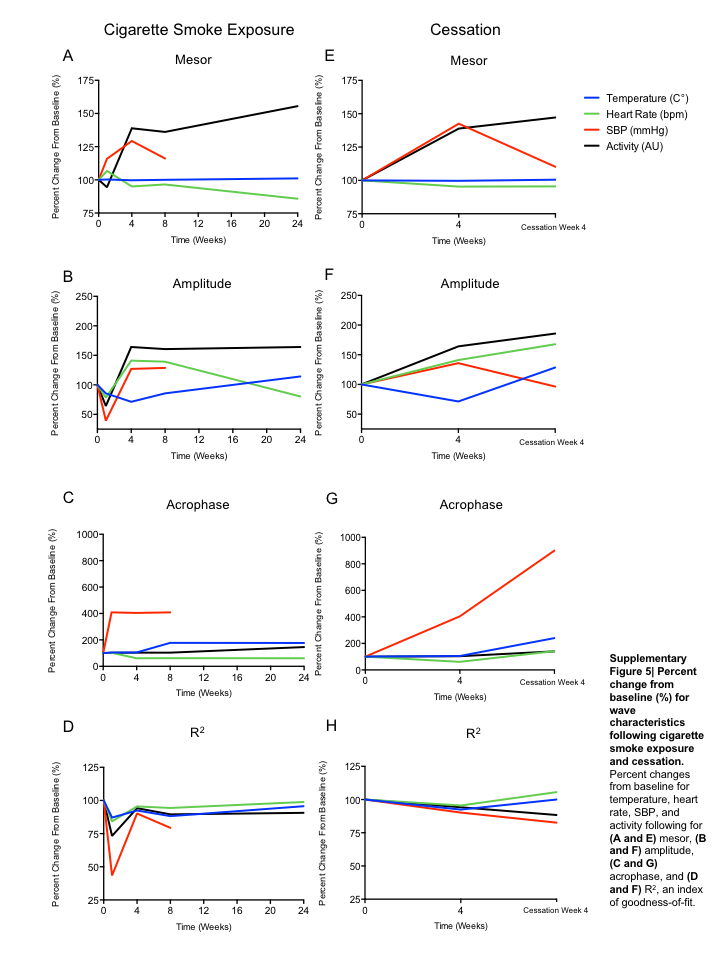

Supplement: Supplementary file 5 [file Image_5.TIFF]
